# Individuality Affects the Efficiency of Basketball Pre-Game Warm-Up on Players’ Performance

**DOI:** 10.3390/sports12120353

**Published:** 2024-12-19

**Authors:** Grigoris Papagiannis, Konstantina Karatrantou, Christos Batatolis, Panagiotis Ioakimidis, Vassilis Gerodimos

**Affiliations:** Department of Physical Education and Sport Science, University of Thessaly, 42100 Trikala, Greecebatatoli@uth.gr (C.B.);

**Keywords:** team sports, vertical jumping ability, sprint, flexibility, heart rate

## Abstract

Pre-game warm-up is integral to athletes’ preparation before a basketball game. The main objectives of this study were to compare specific performance indicators before and immediately after a basketball pre-game warm-up, and examine the individualized players’ responses. The impact of rest intervals after warm-up (9–23 min) was also examined. A total of 20 male basketball players (age: 21.15 ± 2.2 years; body mass: 82.23 ± 10.78 kg; body height: 184.18 ± 7.9 cm) performed a pre-game warm-up and were assessed in selected indicators such as heart rate (HR), flexibility, running speed, and countermovement jump with arm swing (CMJAS) before, immediately after, 9 min and 23 min after warm-up. Immediately after warm-up, HR increased to all players (mean change: 69.78%), while flexibility (mean change: 20.14%) and CMJAS (mean change: 4.95%) increased to the majority of players (except one and two players, respectively). The individualized results regarding speed were conflicting, showing a decrease or increase. However, 9 and 23 min after warm-up, there was a decrease in HR, speed, and CMJAS (*p* < 0.5), while flexibility remained stable (*p* > 0.5) in the total sample, with great change variations among players. In conclusion, it seems that each player’s individuality may affect the warm-up’s efficiency. Thus, it is important to carefully design the pre-game warm-up so that all players will be in the most suitable condition to meet the demands of their competition.

## 1. Introduction

Pre-game warm-up is a common practice followed by coaches in all team sports (i.e., basketball), aiming to physically and psychologically prepare athletes for the competition, to prevent injury, and to maintain or ideally increase players’ performance [1,2,3,4,5]. Although the pre-game warm-up protocol is differently designed by each coach (depending on the sport, the athletes’ level, the season, and the specific game), all protocols share a common structure and philosophy. A pre-game warm-up protocol typically includes low-intensity aerobic exercise, stretching, and sport-specific drills [6]. After a warm-up protocol, various changes occur in the human body such as an increase in muscle temperature, neural conductivity, blood flow, and initial oxygen consumption. At the same time, muscle stiffness is decreased and neuromuscular facilitation is enhanced [6,7,8].

The aforementioned changes, as a result of pre-game warm-up, may immediately influence physical abilities (flexibility, coordination, speed, vertical jump) which significantly determine the overall athletic performance. In the contemporary literature, the number of studies that examined the immediate effect of pre-game warm-up on selected physical abilities in team sports particularly in basketball is quite limited. In more detail, Crowther et al. [9] observed an increase in the vertical jump (6–8.3%) and heart rate (87.0–102.8%) immediately after the basketball pre-game warm-up. In other team sports (volleyball, soccer, handball), previous studies that examined the immediate effects of a pre-game warm-up reported conflicting results. Specifically, Saez de Villarreal et al. [10] observed an increase in vertical jump by 6.96% in volleyball players; whereas, Romaratezabala et al. [3] observed that handball pre-game warm-up did not affect agility and running speed. Other studies in soccer [5,8,11] demonstrated that vertical jump and agility remained stable following a pre-game warm-up, while speed performance improved or decreased. Different factors such as the type, the duration, and the contents of the pre-game warm-up protocol, but mainly the individuality of each athlete, could possibly affect the efficiency of the pre-game warm-up in various physical abilities. For this reason, it is of crucial importance to examine and compare the individual responses of each player to draw valuable information regarding the appropriate design and implementation of an efficient pre-game warm-up for the whole team.

Another crucial aspect of a warm-up lies in the duration of its impact on performance. The time lapse between the start of the game and the entry of substituted players can lead to a decline in their performance in various physical abilities [12]. This is important, especially in basketball, where the regulations of the sport do not allow players on the bench to stand up and re-warm up when they are in substitution. Previous studies in the scientific literature have examined the effect of different time intervals (from 9 to 10 min to 40 min) following the termination of the basketball pre-game warm-up of players’ performance [9,13,14,15]. These studies observed a decrease in the vertical jump [9,13,14,15] and speed performance (increasing the running time during the 20 m sprint test) [14] even within the first 10 min of rest after the pre-game warm-up. It should also be mentioned that the above studies reported an even greater decrease in performance as time of rest/inactivity increased. A previous study that examined flexibility showed that after 9 and 23 min of inactivity, performance in the sit and reach test and back scratch test decreased, though it did not reach the level of statistical significance [15].

Taking all the above into consideration, it is not conclusively established whether a basketball pre-game warm-up protocol has a positive, negative, or neutral impact on athletes’ performance. Although several studies have been conducted comparing how different types of warm-ups affect performance in measured physical abilities [16,17], to the best of our knowledge there is very limited information regarding the immediate effect of a basketball pre-game warm-up on physical abilities [9]. Furthermore, no previous study has examined and compared the efficacy of a basketball pre-game warm-up to each player’s performance separately (individual responses). Thus, the main objective of this study was (a) to evaluate and compare specific performance indicators (flexibility, vertical jump, speed, heart rate) of basketball players before and immediately after a basketball pre-game warm-up, and (b) to examine the individualized players’ responses following the pre-game warm-up in the aforementioned performance indicators. The impact of specific rest intervals after the warm-up (9 and 23 min) on the same performance indicators was also examined during the study. In the scientific literature, there are several pre-game warm-up protocols for basketball. In our study, we chose a widely used protocol in EuroLeague that was designed and implemented by experienced basketball coaches and coaches of physical conditioning. It was hypothesized that the basketball pre-game warm-up would improve the measured indicators in each player (with different percentage changes depending on the player). It was also hypothesized that the rest interval following the pre-game warm-up would decrease the performance of each player (with different percentage changes depending on the player).

## 2. Materials and Methods

### 2.1. Participants

Twenty male adult basketball players (age: 21.15 ± 2.2 years old; body mass: 82.23 ± 10.78 kg; body height: 184.18 ± 7.9 cm) were involved in the current research. All participants were healthy (did not suffer from any chronic disease), did not report the use of any medication, and did not have any injury in the upper and lower limbs for at least 6 months before the commencement of the study. The participants were recruited from four basketball teams from the region of Thessaly in Greece that played at the 3rd division (the five basic player positions at each team were selected, with representation of all playing positions in the final sample). All the participants had 10–11 years of total experience in basketball, participated in basketball training 5 times per week, played one official game per week, and had a day off per week. It should be mentioned that (a) the present study was conducted according to the Declaration of Helsinki, (b) the Institutional Review Board Committee of the local university approved the experimental protocol, and (c) all participants signed the informed consent form before the experiment took place.

### 2.2. Design and Procedures

All training and testing procedures took place on an indoor basketball court. A familiarization session and measurements of anthropometric characteristics (body mass and body height) were performed approximately a week before the study. In more detail, the participants were familiarized with the basketball pre-game warm-up protocol performing two familiarization sessions with the warm-up protocol before the start of the study and the measurements of selected fitness indicators performing familiarization trials for each test before the start of the study. Thereafter, the main experiment was carried out, where the participants performed a pre-game warm-up protocol, and measurements of selected fitness indicators (heart rate, flexibility, running speed, vertical jump) were performed at four different time points (before, immediately after, 9 min, and 23 min after the pre-game warm-up). The measurements, at the four time points, were performed using the following order: heart rate, flexibility, running speed, and vertical jump. The total procedure of the main experiment (simulating real game conditions) included (a) after 5 min rest, 2 min of muscle preparation (where the players under the supervision of basketball coaches performed 2 min of dynamic movements), (b) first measurements (pre-warm-up), (c) basketball pre-game warm-up lasting 30 min (basketball warm-up protocol is analytically presented later), (d) second measurements (immediately after warm-up), (e) 9 min rest after the second measurements (athletes sat on the bench, watching a game on a computer)/third measurements, and (f) 14 min rest after the second measurements (athletes sat on the bench, watching a game on a computer)/fourth measurements (23 min after warm-up). The selection of the two time points (9 and 23 min) following the completion of the pre-game warm-up protocol was based on a previous study from our laboratory, which defined the typical post-warm-up period of inactivity in basketball analyzing 20 games from four professional leagues in Europe [15]. The evaluation of each athlete took place on a single day, with the participants being examined in groups of three in a random order. In the four time points of measurements, the sit and reach test was performed simultaneously for the three basketball players by the three examiners who performed all the measurements. The other tests (10 m sprint and vertical jump) were performed with a difference of 10–20 s among the three players, where during the rest of the first player among trials in each test, the other two players performed their testing trials. The tests were completed within 3 min and 30 s for the three players in each time point. During the warm-up, nine athletes participated along with the three athletes under examination (a total of 12 athletes) to simulate game conditions. The basketball pre-game warm-up protocol was designed and implemented by experienced basketball coaches and coaches of physical conditioning. All measurements were performed by the same examiners, at the same time of the day (9:30–11:30 a.m.) and under similar environmental conditions (26–28 °C).

### 2.3. Basketball Pre-Game Warm-Up

The basketball pre-game warm-up protocol lasted 30 min and consisted of five parts: (1) low-medium intensity aerobic basketball exercise (4 min), (2) stretching exercises (8 min), (3) medium-high intensity running exercises (3 min), (4) high-intensity specific basketball exercises (14 min), and (5) free throws execution (1 min). The warm-up protocol is analytically presented in Table 1.

**Figure 1:**
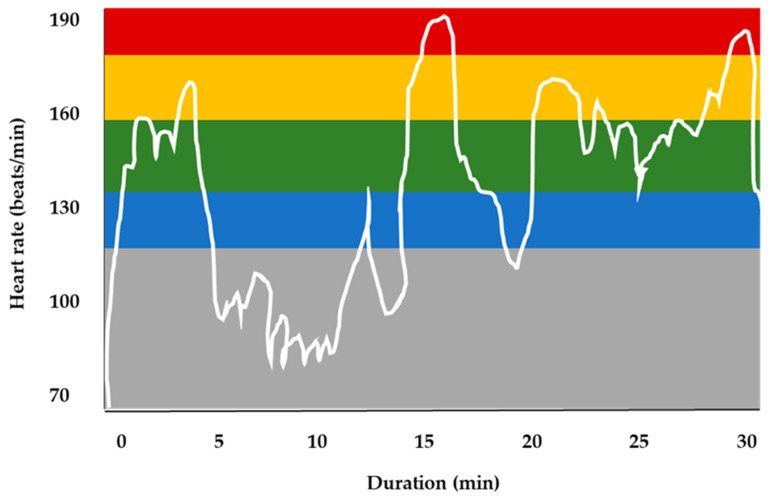
Individual report of an indicative heart rate recording during the warm-up protocol. The grey zone in the figure indicates an intensity level below 60% of age-predicted maximum heart rate (HR_max_), the blue zone an intensity level 60–69% of HR_max_, the green zone an intensity level 70–79% of HR_max_, the yellow zone indicates an intensity level 80–89% of HR_max_, and the red zone an intensity level ≥ 90% of HR_max_.

During the warm-up protocol, a real-time monitoring of the players’ heart rate using the Polar Team Solution system (Science Technologies, Kempele, Finland) was performed. An indicative report (from an athlete) of heart rate recording during the warm-up protocol is graphically presented in Figure 1.

### 2.4. Measures

✓ Heart rate: Heart rate was measured using a specialized heart rate monitor Polar H10 (Polar, Kempele, Finland). The participants wore a chest strap with the transmitter positioned on top. The transmitter was connected to a tablet application, providing the athlete’s heart rate at any given moment.
✓ Flexibility: The sit and reach test was performed to evaluate flexibility using a Flex-Tester box (Novel Products Inc, Rockton, IL), as previously described by ACSM [18]. The test was repeated three times with a rest period of 10 s and the best score (in cm) was recorded [18]. The sit and reach test showed excellent test-retest reliability (ICC = 0.98 − 0.99) [19,20] and inter-rater reliability (ICC = 0.97) in team sport athletes [20].
✓ Running speed: 10 m sprint in a straight line was measured using four photocells (Velleman PEM10D, Gavere, Belgium) connected to an electronic timer (Chronopic, Chronojump Bosco System, Barcelona, Spain). During the test, athletes started from an upright position with one foot forward. Each athlete performed two sprints with a rest period of 1 min in between, with the better performance (time in s) being recorded. The 10 m sprint test showed good test-retest reliability (ICC = 0.83) [21] and inter-trial reliability (ICC = 0.81) [22] in young team sport athletes.
✓ Vertical jumping ability: Vertical jumping ability was measured according to the countermovement jump test with arm swing (CMJAS) using the Optojump Next system (Microgate, Bolzano, BZ, Italy), as previously described by Gerodimos et al. [23]. Each athlete performed two jumps with a 1 min rest interval in between and the best performance (height in cm) was recorded. The CMJAS showed high test-retest reliability (ICC = 0.82 − 0.93) [24,25] and inter-trials reliability (ICC = 0.97) [24] in young athletes.

### 2.5. Statistical Analysis

All statistical analyses were performed using IBM SPSS Statistics v.26 software (IBM Corporation, Armonk, New York, NY, USA). The normality of data was examined using the Shapiro–Wilk test (all variables followed the normal distribution). The assumption of sphericity was examined using Mauchly’s test of sphericity (sphericity assumed to all variables). One-way repeated measures analyses of variances (ANOVAs) were used, followed by Sidak’s pairwise comparisons, to examine possible differences among the four time points. Furthermore, bivariate correlation analyses were used to examine possible associations among percentage changes in different time points and performance pre- and immediately after warm-up. All values are presented in the form of mean ± SD and the level of significance was set at *p* < 0.05.

## 3. Results

### 3.1. Flexibility (Sit and Reach Test)

**Figure 2:**
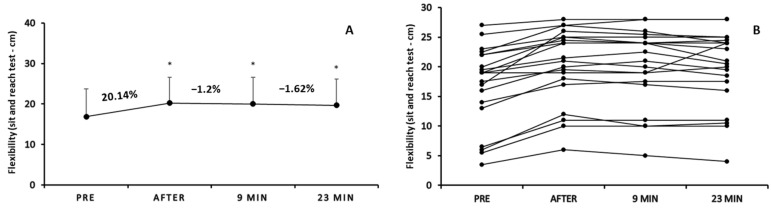
Mean score of flexibility (sit and reach test) pre, immediately after, 9 min, and 23 min after warm-up protocol (**A**) and individualized changes (per participant) in flexibility among the four time points (**B**). * *p* < 0.05 vs. pre-warm-up measurement.

One-way repeated measures analysis of variance demonstrated a significant time effect on flexibility (F3,57 = 30.688; *p* < 0.05; Figure 2A). More specifically, the sit and reach score was significantly (*p* < 0.05) improved immediately after (20.27 ± 6.31 cm), 9 min after (20.02 ± 6.54 cm), and 23 min after pre-game warm-up (19.70 ± 6.51 cm) compared to pre-warm-up score (16.87 ± 6.83 cm). However, there were no significant changes immediately after, 9 min, and 23 min after warm-up time points (*p* > 0.05).

After analyzing and presenting the results individually (Figure 2B), we observed that immediately after the pre-game warm-up, the performance in the sit and reach test was improved for all athletes except one where the score remained unchanged (mean % change: 20.14%; range of change: from 0% to 81.81% depending on the participant). The individual results drawn on the comparisons between immediately after warm-up and 9 min after (mean % change: −1.23; range of change: from −16.66 to 4.65%) as well as between 9 min after and 23 min after (mean % change: −1.62; range of change: from −12.5% to 26.31%) were conflicting. It was highlighted that some basketball players’ performance in the sit and reach test remained stable, while there were also cases of an increase and a decrease in performance.

Bivariate correlation analyses, in flexibility, demonstrated (a) significant negative correlation between the percentage change immediately after and the percentage change 9 min after (r = −0.552; *p* = 0.01), (b) significant positive correlation between the flexibility performance values pre-warm-up and the percentage change 9 min after (r = 0.540; *p* = 0.014) and between flexibility performance values immediately after warm-up and the percentage change 9 min after (r = 0.520; *p* = 0.019), and (c) significant negative correlation between the initial flexibility performance values and the percentage change immediately after warm-up (r = −0.766; *p* < 0.001).

### 3.2. Running Speed (10 m Sprint Test)

**Figure 3:**
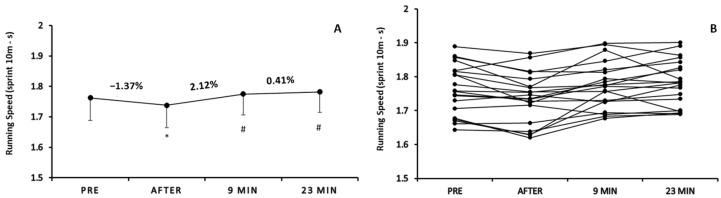
Mean score of running speed (10 m sprint test) pre-, immediately after, 9 min, and 23 min after warm-up protocol (**A**) and individualized changes (per participant) in running speed among the four time points (**B**). * *p* < 0.05 vs. pre-warm-up measurement; # *p* < 0.05 vs. immediately after warm-up measurement.

One-way repeated measures analysis of variance demonstrated a significant time effect on running speed (F3,57 = 12.425, *p* < 0.05; Figure 3A). Running time (during sprint test) was significantly decreased (*p* < 0.05) immediately after warm-up (1.73 ± 0.07 s) vs. pre-warm-up (1.76 ± 0.07 s), while increased at 9 min (1.77 ± 0.06 s) and 23 min after warm-up (1.78 ± 0.06 s) vs. immediately after warm-up (*p* < 0.05).

After analyzing and presenting the results individually (Figure 3B), we observed inconsistent results. For several players, the time needed to sprint a distance of 10 m was decreased but there were cases where this time was increased immediately after warm-up. The mean percentage change was −1.37% ranging from −4.54% to 2.14%. Similarly, the individual results drawn on the comparisons between immediately after warm-up and 9 min after (mean % change: 2.12%; range of change: from −1.57% to 7.98%) as well as between 9 min after and 23 min after (mean % change: 0.41; range of change: from −4.57% to 2.98%) also suffered from high variance. It was highlighted that some basketball players’ running time (during sprint test) was increased, while in others was decreased.

Bivariate correlation analyses, running speed, showed (a) significant negative correlation between the percentage change immediately after and the percentage change 9 min after (r = −0.550; *p* = 0.01) as well as between the percentage change 9 min after and the percentage change 23 min after (r = −0.701; *p* < 0.001), (b) no significant correlations among immediately post-warm-up sprint performance values or pre-warm-up sprint performance values and the percentage changes throughout the rest period of 9 and 23 min (*p* > 0.05) and (c) no significant correlation between initial sprint performance values and percentage change immediately after warm-up (*p* > 0.05).

### 3.3. Vertical Jump (Countermovement Jump Test with Arm Swing—CMJAS)

**Figure 4:**
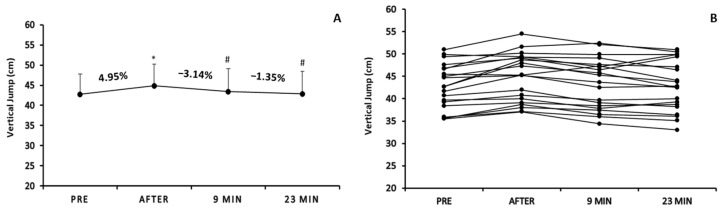
Mean score of vertical jumping ability (countermovement jump with arm swing—CMJAS) pre-, immediately after, 9 min, and 23 min after warm-up protocol (**A**) and individualized changes (per participant) in vertical jumping ability among the four time points (**B**). * *p* < 0.05 vs. pre-warm-up measurement; # *p* < 0.05 vs. immediately after warm-up measurement.

One-way repeated measures analysis of variance demonstrated a significant time effect on the vertical jump (F3,57 = 10.920, *p* < 0.05; Figure 4A). In more detail, CMJAS was significantly (*p* < 0.05) higher immediately after the warm-up (44.48 ± 5.33 cm) vs. pre-warm-up (42.72 ± 5.3 cm), but significantly decreased at 9 min (43.43 ± 5.64 cm) and 23 min after warm-up (42.84 ± 5.64 cm) compared to immediately after warm-up (*p* < 0.05).

After analyzing and presenting the results individually (Figure 4B), we observed that immediately after the pre-game warm-up, the performance in CMJAS was improved for the majority of athletes, except for two where the score was slightly decreased (mean % change: 4.95; range of change: from −1% to 14.05% depending on the participant). The individual results drawn on the comparisons between immediately after warm-up and 9 min after showcased that for all the participants (except for two athletes), the vertical jump decreased (mean % change: −3.14%; range of change: from −7.27% to 4.41%). Additionally, the individual results drawn on the comparisons between 9 min after and 23 min after warm-up showcased that for all the participants (except for two athletes: one athlete increased his performance and one showed no effect), the vertical jump decreased (mean % change: −1.35%; range of change: from −7.2% to 6.23%).

Bivariate correlation analyses, in the vertical jump, showed (a) significant negative correlation between the percentage change immediately after warm-up and the percentage change 23 min after warm-up (r = −0.507; *p* < 0.05), (b) no significant correlations among immediately post-warm-up vertical jump values or pre-warm-up vertical jump values and the percentage changes throughout the rest period of 9 and 23 min (*p* > 0.05), and (c) no significant correlation between initial vertical jump performance values and percentage change immediately after warm-up (*p* > 0.05).

### 3.4. Heart Rate

**Figure 5:**
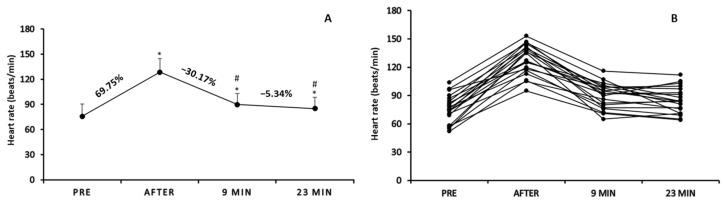
Mean score of heart rate pre-, immediately after, 9 min, and 23 min after warm-up protocol (**A**) and individualized changes (per participant) in heart rate among the four time points (**B**). * *p* < 0.05 vs. pre-warm-up measurement; # *p* < 0.05 vs. immediately after warm-up measurement.

One-way repeated measures analysis of variance demonstrated a significant time effect on heart rate (F3,57 = 141.502, *p* < 0.05; Figure 5A). Heart rate was significantly higher immediately after (128.6 ± 16.01 beats/min), 9 min (89.8 ± 13.36 beats/min), and 23 min after warm-up (85.0 ± 13.73 beats/min) than pre-warm-up (75.75 ± 14.88 beats/min) (*p* < 0.05). Additionally, heart rate value was significantly lower 9 min and 23 min after warm-up compared to immediately after warm-up (*p* < 0.05).

Concerning heart rate, when the results were analyzed and presented individually (Figure 5B), we found that all participants showed increased heart rate immediately after warm-up compared to pre-warm-up (mean % change: 69.78%; range of change: from 22.91% to 149.09% depending on the player). The individual results drawn on the comparisons between immediately after and 9 min after warm-up demonstrated that the heart rate was decreased for all participants (mean % change: −30.17%; range of change: from −16.1% to −51.85% depending on the players). Finally, conflicting results were observed on the measurements from 9 min to 23 min of inactivity as the heart rate of several players remained stable, while for others it was either increased or decreased (mean % change: −31.64%; range of change: from −33.64% to 16.66%).

Bivariate correlation analyses, in heart rate, revealed (a) significant negative correlation between the percentage change immediately after warm-up and the percentage change 9 min after warm-up (r = −0.724; *p* < 0.001) as well as between the percentage change 9 min after and the percentage change 23 min after (r = −0.466; *p* < 0.05), (b) no significant correlations among immediately post-warm-up heart rate values or pre-warm-up heart rate values and the percentage changes throughout the rest period of 9 and 23 min (*p* > 0.05), and (c) significant negative correlation between the initial heart rate values and the percentage change immediately after warm-up (r = −0.886; *p* < 0.001).

## 4. Discussion

Our results demonstrated that heart rate increased in all players, by an average of 69.78%, immediately after the basketball pre-game warm-up protocol. However, the individualized analysis showed great variation in the percentage change in heart rate among players ranging from 22.91% to 149.09%, reinforcing the view that the individuality of each player affects the heart rate response following a basketball pre-game warm-up. The results of previous studies [9,14], which measured basketball players’ heart rates immediately after a pre-game warm-up, were found to be aligned with our findings as heart rate increased in both cases by an average of 94.9% and 159.8%, respectively. The increase in heart rate observed in this and previous studies may be linked to the fact that a warm-up activates the sympathetic function of the cardiovascular system, which increases the cardiac output and consequently the heart rate [26]. Furthermore, our findings showed an increase in flexibility immediately after a basketball pre-game warm-up for the vast majority of players (except one player where the flexibility remained stable) by an average of 20.14% with great however individual variations in the percentage change among players ranging from 0 to 81.81%. The increased flexibility observed in this and previous studies may be attributed to the fact that the increased muscle temperature due to the warm-up reduces muscle resistance to tension, increases joint range of motion, and enhances muscle relaxation speed [27,28]. The great individual variations in the percentage changes of flexibility and heart rate immediately after the warm-up may be attributed to the initial performance level in these indicators and the general physical conditioning level of each player. This notion has been strengthened by the results of our study that showed significant negative correlation between the initial flexibility or heart rate values and the percentage change immediately after the warm-up. In more detail, the basketball players with higher initial flexibility and heart rate values showed lower percentage change immediately after the pre-game basketball warm-up protocol.

The next important indicator examined in the current study was running speed. It is evident that running speed greatly determines the performance of basketball players as during the game they need to run as fast as possible to cope with both offense and defense [29]. Our study highlighted that for the total sample of basketball players, the time needed to sprint a 10 m distance immediately after a warm-up was decreased by an average of −1.37%. However, the individualized results regarding running speed were conflicting, showing either a decrease or increase in running time (% change: from −4.54 to +2.14). The great individual variations in running speed changes immediately after the warm-up may be attributed to the initial speed performance level (although in our study no significant correlation between initial sprint performance values and percentage change immediately after the warm-up were observed) and the general physical conditioning level of each player as well as to the different receptivity (physical and mental) of each player to the warm-up stimulus received. Previous studies that examined the effect of a pre-game warm-up on speed performance in other team sports (handball, soccer) also showed conflicting results. Specifically, Romaratezabala et al. [3] showed that a handball warm-up had no immediate effect on athletes’ performance on the 5 m and 15 m sprint tests, while other studies in soccer showed either a decrease or increase in speed performance immediately after a warm-up by an average of 3.62–4.51% [5,11]. The conflicting results among studies regarding speed performance may be attributed to different factors such as the type, intensity, and duration of the warm-up protocol used as well as the players’ characteristics.

An additional important indicator that was measured in the present study is the vertical jump. Our findings demonstrated an increase in CMJAS in the vast majority of basketball players immediately after the pre-game warm-up by an average of 4.95% (except for two players who showed a very small drop in CMJAS performance). However, the individualized analysis showed variation in the percentage change in CMJAS among players ranging from −1% to 14.05%, reinforcing the impact of individuality in the performance responses following a basketball pre-game warm-up protocol. A previous study [9] that assessed the vertical jump of two athletes before and after a warm-up observed an increase in performance for both of them by 8.3% and 6%, respectively. Additionally, a previous study conducted in volleyball showed significant improvement in different vertical jump tests (CMJ, DJ, CMJ with loads) by 6.96%, 4.49%, and 10.9% after a pre-game warm-up [10]. Different conclusions are drawn in the field of soccer, as the analysis of data from three studies suggests that the vertical jump before and after warm-up remains unchanged [5,8,11]. This difference may be attributed to the nature of the soccer pre-competitive warm-up, which does not include jumping movements to activate the stretch-shortening cycle to such an extent as to increase performance.

As mentioned earlier, the course of the competition is also determined by the effect of inactivity on athletes’ performance when they are not competing. Research has highlighted that the effect of warm-up on players decreases while they are inactive, and thus their performance in different physical fitness indicators decreases as time passes [12]. In sports such as soccer or volleyball, athletes are allowed to warm up again with specific exercises on the sidelines before entering the game, thus addressing this performance decline. However, in basketball, regulations do not allow players on the bench to stand up when they are in substitution, making the maintenance of possible performance improvement following the termination of the warm-up an important aspect. Our results demonstrated that after 9 and 23 min of rest following a warm-up, there was a decrease in HR, speed, and CMJAS performance, while flexibility remained stable in the total sample. Previous studies also reported similar results showing a decrease in performance following the basketball pre-game warm-up. Regarding vertical jump performance as an instance, a decrease of 3.8% to 13% was observed in the first 10 min of rest after a basketball pre-game warm-up, while at 20 min the decrease ranged from 7.3% to 20% [9,13,14,15]. In terms of speed, in the 20 m sprint, an increase of 3.9% to 6.3% of running time was observed at 10 min and 40 min of rest after a pre-game warm-up. Specifically, in the 0 to 10 m split time, the increase in running time ranged from 5% (in the first 10 min) to 8.5% (in 40 min), and in the 10 to 20 m split time, the average increase was 3.4% [14]. Regarding flexibility, our results are consistent with the study by Kapnia and her colleagues [15] in which flexibility (although it decreased) did not significantly differ at 9 and 23 min after a warm-up compared to immediately after a warm-up. However, in our study, the individualized results showed great change variations among players as some maintained stable performance while others experienced a decrease or increase in physical abilities 9 and 23 min following the basketball pre-game warm-up. The initial performance level of each player as well as the magnitude of percentage change immediately after the basketball warm-up could possibly affect the percentage change during the rest periods following the termination of the warm-up. Our results support this notion, demonstrating that basketball players with greater percentage change immediately after the warm-up showed lower percentage change after rest periods.

The overall decrease in athletes’ performance after periods of inactivity following the competitive warm-up can be attributed to various factors. Firstly, body temperature drop due to inactivity is crucial as it affects the rate and speed of muscle contraction and reduces the rate of energy production, resulting in less effective explosive movements relying on these mechanisms [30]. From a biochemical perspective, performance may be influenced by plasma glucose concentration levels as a decrease in these levels due to inactivity limits energy reserves for intense activities [14]. Additionally, the seated position may also negatively affect the athletes’ performance, especially regarding the vertical jump. Studies’ results in this area have shown that the vertical jump of athletes who remained standing for a period of inactivity after a competitive warm-up decreased less compared to those who sat on the bench [13]. During the last decades, however, coaches and researchers examined different alternative methods to eliminate the negative impact of inactivity following the basketball pre-game warm-up. Coaches have sometimes applied exercises on a cycle ergometer at the edge of the bench for specific players who are on substitution attempting to maintain stable body and muscle temperature. Another approach could be to activate the muscles from a seated position using isometric exercises or elastic bands. However, a study involving athletes performing isometric exercises on the leg muscles and mobilizing the back did not report positive results [13]. The Dri-Fit thermal clothing is an alternative way of maintaining athletes’ temperature; however, research examining this method also did not showcase promising findings [15].

This study has some limitations that could affect the generalization of its findings. First, the results of this study are limited to young male adult basketball players of the 3rd division and to the use of the specific pre-game basketball warm-up protocol. Future studies could examine the efficacy of the specific pre-game basketball warm-up protocol in basketball players with different characteristics (i.e., female players, players of other divisions, players during the developmental years). Future studies could also compare the efficacy of the selected pre-game basketball warm-up protocol with other protocols used in the scientific literature. An additional significant limitation of the present study is the measured time points, where the third set of testing (9 min after the basketball warm-up protocol) could affect the fourth set of testing (23 min after the basketball warm-up protocol), rewarming up basketball players. Furthermore, although our sample consists of players from all basketball positions (point guard, shooting guard, small forward, power forward, and center), the sample of players per position is too small to draw safe results regarding possible differences in responses among players from different positions. However, future studies with a greater sample per basketball could examine differences among the various playing positions. Moreover, though in our study we found some significant correlations among initial or immediately after warm-up performance values of basketball players and different percentage changes, the sample of the present study is too small to draw safe conclusions on this topic. Future studies with a greater sample could yield safer conclusions in this topic by running different correlations analyses to better explain the great variations in individual responses. Finally, although in our study we examined the impact of rest intervals after a warm-up (9–23 min) in all basketball players, we did not examine the efficacy of an alternative method to eliminate the negative impact of rest following the basketball pre-game warm-up. Future studies could examine and compare the efficacy of different alternative methods to eliminate the negative impact of inactivity following the basketball pre-game warm-up, examining simultaneously the individual responses of basketball players.

## 5. Conclusions

It seems that a carefully designed basketball pre-game warm-up may positively immediately affect different physical fitness indicators of the total team; however, the individual response of each player influences the efficacy of warm-up. For this reason, it is of crucial importance for coaches to carefully design the basketball pre-game warm-up protocol and systematically examine its efficiency for all players’ performance. Furthermore, our results support previous findings that the rest period following the pre-game warm-up negatively affects players’ performance. Thus, it would be of great importance to conduct research examining various practices capable of maintaining the changes caused by a warm-up, so that athletes perform at their maximum upon entering, without needing adjustment time.

## Figures and Tables

**Table 1:** Basketball pre-game warm-up protocol (total duration: 30 min).

Part 1: Low-medium intensity aerobic basketball exercise (duration: 4 min) Lay-ups (the numbered coloured circles in the figure below represent the basketball players) ** 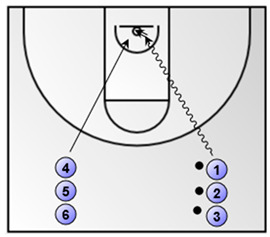 **	
Part 2: Static and Dynamic stretching exercises (duration: 8 min) ✓ Upper Body: overhead shoulder stretch, rotation core stretch, overhead triceps stretch with side bent, forward and backward arm rotation, up and down arm swing, lateral arm swing. ✓ Lower Body: quadricep stretch, hamstring stretch, glute stretch, abductors stretch, calf stretch, lateral leg swing, front leg swing.	
Part 3: Medium-high intensity running exercises (duration: 3 min) Exercise 1. Hops, sprint until half line and sprint back (2 reps). Exercise 2. Low skipping until free throw line, close-out until half line and backpedal until baseline (2 reps). Exercise 3. Butt kicks until free throw line, close-out until half line and defensive slide until baseline (2 reps).	
Part 4: High intensity specific basketball exercises (duration: 14 min)	
Exercise 1. 1 vs. 1, pass and close-out and 1 vs. 1 (duration: 3 min; the numbered coloured circles in the figure below represent the basketball players). * 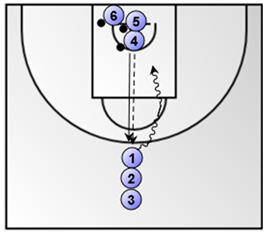 *	Exercise 2. a. change in direction and shot/drive, b. change in direction and low post play (duration: 5 min; the numbered coloured circles in the figure below represent the basketball players). 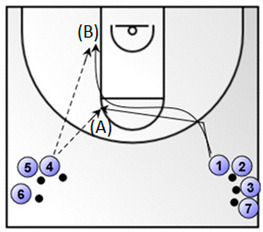
Exercise 3. Shooting after dribble from the top and corners (duration: 3 min; the numbered coloured circles in the figure below represent the basketball players). * 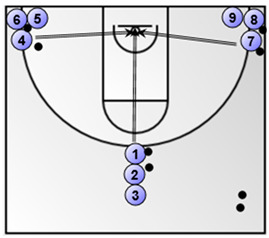 *	Exercise 4. Lay-up without dribble (duration: 3 min; the numbered coloured circles in the figure below represent the basketball players and the circle with the letter C represent the basketball coach). * 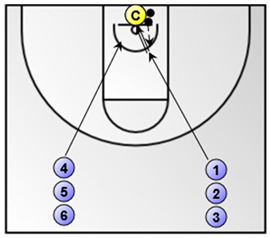 *
Part 5: Free Throws (duration: 1 min)	

## Data Availability

Data are unavailable due to privacy or ethical restrictions.
